# Spoken Language Skills in Children With Bilateral Hearing Aids or Bilateral Cochlear Implants at the Age of Three Years

**DOI:** 10.1097/AUD.0000000000001092

**Published:** 2021-07-13

**Authors:** Taina T. Välimaa, Sari Kunnari, Antti A. Aarnisalo, Aarno Dietz, Antti Hyvärinen, Jaakko Laitakari, Sari Mykkänen, Satu Rimmanen, Jaakko Salonen, Ville Sivonen, Tanja Tennilä, Teija Tsupari, Sari Vikman, Nonna Virokannas, Päivi Laukkanen-Nevala, Anna-Kaisa Tolonen, Krista Tuohimaa, Heikki Löppönen

**Affiliations:** 1 Research Unit of Logopedics and Child Language Research Center, University of Oulu, Oulu, Finland; 2 University of Helsinki, Helsinki, Finland; 3 Department of Otorhinolaryngology–Head and Neck Surgery, Helsinki University Hospital, Helsinki, Finland; 4 Department of Otorhinolaryngology—Head and Neck Surgery, Kuopio University Hospital, Kuopio, Finland; 5 Department of Otorhinolaryngology–Head and Neck Surgery, Oulu University Hospital, Oulu, Finland; 6 Department of Otorhinolaryngology—Head and Neck Surgery, Tampere University Hospital, Tampere, Finland; 7 Department of Otorhinolaryngology–Head and Neck Surgery, Turku University Hospital, Turku, Finland; 8 Department of Otorhinolaryngology, Institute of Clinical Medicine, University of Eastern Finland, Kuopio, Finland.

**Keywords:** Bilateral cochlear implant, Bilateral hearing aid, Children, Lexicon, Outcomes, Phonology, Spoken language comprehension

## Abstract

Early hearing aid (HA) fitting and cochlear implants (CIs) aim to reduce the effects of hearing loss (HL) on spoken language development. The goals of this study were (1) to examine spoken language skills of children with bilateral HAs and children with bilateral CIs; (2) to compare their language skills to the age-norms of peers with normal hearing (NH); and (3) to investigate factors associated with spoken language outcomes.

**Design:**

Spoken language results of 56 Finnish children with HL were obtained from a nationwide prospective multicenter study. Children with HL comprised two groups: children with mild-to-severe HL who used bilateral HAs (BiHA group, n = 28) and children with profound HL who used bilateral CIs (BiCI group, n = 28). Children’s spoken language comprehension, expressive and receptive vocabulary, and phonological skills were compared with normative values of children with NH at the age of three years. Odds ratio (OR) was calculated to compare proportions of children below age-norms in BiHA and BiCI groups. Factors associated with spoken language outcomes were modeled with analysis of covariance.

**Results:**

At the age of 3 years, 50%–96% of children with HL performed 1 SD or more below the mean of the normative sample of age-peers with NH in spoken language skills, depending on the language domain. Receptive vocabulary and phonological skills were the most vulnerable language domains. In receptive vocabulary, 82% of the children in the BiHA group and 50% of the children in the BiCI group scored 1 SD or more below the normative mean. The BiHA group was 4.4 times more likely to have poorer receptive vocabulary than the BiCI group. In phonological skills, 96% of children in the BiHA group and 60% of the children in the BiCI group scored 1 SD or more below the normative mean. The BiHA group was 18.0 times more likely to have poorer phonological skills than the BiCI group. The analysis of covariance models showed that unaided pure-tone average, PTA_0.5–4 kHz_, had a significant effect on spoken language comprehension in the BiHA group. For the BiCI group, age at HL diagnosis and age at CI activation had a significant effect on expressive vocabulary. High maternal level of education had a significant effect on language comprehension and expressive vocabulary and female gender on phonological skills.

**Conclusions:**

At the age of 3 years, especially receptive vocabulary and phonological skills caused difficulties for children with HL showing also considerable individual variation. Children with bilateral HAs seemed to be more likely to have poorer receptive vocabulary and phonological skills than children with bilateral CIs. A variety of factors was associated with outcomes in both groups. Close monitoring of spoken language skills of children with HL is important for ensuring similar opportunities for all children with HL and timely intervention, when needed.

## INTRODUCTION

Hearing loss (HL) is a common disorder with a prevalence of 1 to 3 in 1000 newborns (Fortnum et al. 2001; Russ et al. 2003). HL can be congenital or acquired and can range in severity from mild (21–40 dB HL) through moderate (41–70 dB HL) to severe-profound (≥70 dB HL) calculated as the average better ear hearing level without devices at the frequencies of 0.5, 1, 2, and 4 kHz (British Society of Audiology 2011). Approximately 95% of children with HL are born to parents with normal hearing (NH), with spoken language as the communication mode, no previous history of HL (Mitchell & Karchmer 2004), and no knowledge of sign language. Therefore, parental expectations for spoken language development are often high. Earlier research has shown that HL poses a risk for spoken language development (Wake et al. 2005; Duchesne et al. 2009; Fitzpatrick et al. 2011; Boons et al. 2012a; Ching et al. 2013; Tomblin et al. 2015; Dettman et al. 2016). For children with NH, a persisting language delay is highly predictive of poor reading and academic outcomes (Scarborough 2001; Zambrana et al. 2014). Thus, children with HL are at risk of poor literacy skills (Geers et al. 2008), academic skills (Geers et al. 2003, 2008), and social development (Most et al. 2010). One obvious reason is that children with HL who use hearing aids (HAs) or cochlear implants (CIs) perceive speech signals that are distorted or degraded and often quieter, which causes a child’s ability to receive important acoustic cues to fall to near or below threshold (Moore 2007; Wilson & Dorman 2008). This is magnified in noisy environments. If the intervention needs of these children are not met at an early age, the consequences may be far-reaching and society’s costs substantial. In the present research, we focus on spoken language skills of preschool-aged children with mild-to-profound HL and factors associated with it.

## SPOKEN LANGUAGE SKILLS IN CHILDREN WITH HEARING AIDS OR COCHLEAR IMPLANTS

Recent studies on spoken language outcomes of children with mild-to-profound HL who use HAs have shown great variability in children’s performance. Despite newborn hearing screening and early diagnosis, many children with HAs still fall within the lowest quarter or below age-norms of peers with NH on several measures of spoken language before school-age and during elementary school (Wake et al. 2005; Yoshinaga-Itano et al. 2010; Fitzpatrick et al. 2011; Ching et al. 2013; Tomblin et al. 2015; Yoshinaga-Itano et al. 2018). Research even suggests that if children with HL are compared with children with NH matched on socioeconomic status, the difference between children with HL and NH is greater than when compared to general age-norms (Tomblin et al. 2015). Children with HAs show persisting delays especially in vocabulary, phonology, and morphology (Mayne et al. 1998a,b; Baudonck et al. 2010; Johnson & Goswami 2010; Moeller et al. 2010; Baudonck et al. 2011; Koehlinger et al. 2013; Lederberg et al. 2013; Tomblin et al. 2015; Hammer & Coene 2016; Halliday et al. 2017). Morphological development may be delayed even in adolescence (Delage & Tuller 2007; Huysmans et al. 2014). On the other hand, improvement in spoken language skills during the early school years may yield improvement also in literacy skills (Tomblin et al. 2020). These findings highlight the need to monitor children’s language skills over time to prevent potential delays in spoken language and literacy skills.

For children with profound HL, unilateral CIs have clearly improved spoken language development, but children’s language profiles are heterogeneous (Boons et al. 2012a; van Wieringen & Wouters 2015). Some children perform within age-norms in phonological, lexical, morphological, and syntactic skills in preschool or during elementary school (Duchesne et al. 2009; Fulcher et al. 2012; Leigh et al. 2013; Dettman et al. 2016). Recent reports show, however, that up to 60% of children with CIs lag behind age-peers at least on some measures of spoken language (Duchesne et al. 2009; Yoshinaga-Itano et al. 2010; Percy-Smith et al. 2012; Dettman et al. 2016). Acquisition of receptive and expressive vocabulary has been found to be delayed even at adolescence (Duchesne et al. 2009; Yoshinaga-Itano et al. 2010; Boons et al. 2012a; Percy-Smith et al. 2012; Davidson et al. 2014; Lund 2016; Välimaa et al. 2018). In addition, phonological and phonetic skills (Baudonck et al. 2010; Baudonck et al. 2011), and morphological and syntactic skills have all induced difficulties for children with CIs (Boons et al. 2013; Hammer & Coene 2016). Such heterogeneous findings emphasize the need for early and systematic assessment of spoken language development to provide timely and adequate speech and language intervention, if needed.

## FACTORS ASSOCIATED WITH SPOKEN LANGUAGE DEVELOPMENT

### Auditory Factors

Understanding the many auditory, child-related and environmental factors that contribute to spoken language outcomes play a crucial role in intervention planning. Newborn hearing screening, early HL diagnosis, and early HA fitting have greatly improved spoken language outcomes of children with HAs as compared to the era before screening (Yoshinaga-Itano et al. 2010; Korver et al. 2010; Pimperton & Kennedy 2012; Yoshinaga-Itano et al. 2017, 2018). Early HA fitting is reported to have a positive influence on speech perception and speech production outcomes, especially at 3 to 5 years of age (Sininger et al. 2010). However, another study found only a weak association even at that age (Ching et al. 2013). Furthermore, some studies show that age of HA fitting, and language and communication outcomes may not be associated with each other at the age of 6 years (Tomblin et al. 2015) or at the age of 7 to 8 years (Wake et al. 2005). Indeed, thanks to newborn hearing screening, there may be less variability in the ages at which children are fit with HAs. This may lead to a weaker association between age at HA fitting and language outcomes compared to the era before newborn screening, when children were diagnosed and fitted with HAs later in toddlerhood. The association between age at HA fitting and spoken language outcomes may also be weaker at the age of 6 to 8 years compared to the age of 3 to 5 years, since children’s spoken language skills continue to develop with consistent HA use (Wake et al. 2005; Tomblin et al. 2015). For children with CIs, early age of implantation compared to later ages is clearly associated with better spoken language outcomes (Connor et al. 2006; Geers et al. 2016; Dettman et al. 2016; Yoshinaga-Itano et al. 2018). Children implanted at around the age of 1 year or younger may better catch-up with their NH peers compared to children implanted later.

In addition to HA fitting age, HA use is an important auditory factor to consider for children with HAs. Recent reports show that some children do not use their HAs systematically (Walker et al. 2015b) and that nonsystematic HA use has a negative impact on speech and language development (Walker et al. 2015a; Tomblin et al. 2015). Studies on the reasons for systematic HA use have shown that especially mothers’ higher level of education is associated with children’s systematic HA use (Marnane & Ching 2015; Walker et al. 2015b). Additionally, high maternal self-efficacy (the belief in one’s ability to perform a task successfully) in device care and maintenance has been associated with higher parental involvement in developing their child’s speech and language skills (Desjardin 2005). Furthermore, even though a majority of children with CIs seem to use their CIs systematically (i.e., >8 or 9 hours daily), not all do so (Easwar et al. 2016; Busch et al. 2017). Full-time CI use has been shown to be associated with better spoken language skills at the age of 3 years, but it is not necessarily established for all children with CIs by the age of three years (Park et al. 2019). Inconsistent CI use has been associated with, for example, repeated disconnections between the external transmission coil and the internal device (Easwar et al. 2016). Without doubt, degree of HL and aided speech audibility with HAs, as measured by the speech intelligibility index (SII), influence spoken language development; the more severe the HL and the poorer the aided speech audibility, the more negative the impact (Wake et al. 2005; Stiles et al. 2012; Koehlinger et al. 2013; Tomblin et al. 2015). These findings point out the complex intercorrelations of auditory and environmental factors such as systematic HA/CI use, maternal level of education and self-efficacy, and parental involvement that are all associated with spoken language outcomes.

### Child-Related and Environmental Factors

Clearly, additional disabilities, for example, features of autism spectrum disorder, neurological and developmental disorders, familial language impairment, and even male gender are among the major child-related factors negatively associated with spoken language outcomes of children with HAs or CIs (Hawker et al. 2008; Boons et al. 2013; Ching et al. 2013; Ching & Dillon 2013; de Hoog et al. 2015). Of environmental factors, especially high maternal level of education and socioeconomic status of the family, and quantity and good quality of caregivers’ linguistic input, have positively influenced speech and language development of children with HAs as well as of children with CIs (Niparko et al. 2010; Fitzpatrick et al. 2011; Ching et al. 2013; Ambrose et al. 2015; Halliday et al. 2017; Nittrouer et al. 2020).

## DIFFERENCES BETWEEN CHILDREN WITH HEARING AIDS AND COCHLEAR IMPLANTS

Some studies have sought to compare spoken language profiles of children with mild-to-profound HL who receive HAs to those of children who receive CIs. The few studies have produced inconsistent results. Sininger et al. (2010) found that CI use was associated with better speech perception, speech production, and language outcomes in preschool-aged children with HL. In their study, 16 children were fitted with mainly unilateral CIs at the median age of 28.5 months (minimum 12.78, maximum 76.48, 12 unilateral, 4 bilateral), while 28 children with mild-to-profound HL continued to use HAs. Fitzpatrick et al. (2011) found no significant differences in receptive and expressive language skills between children with severe to profound HL who used CIs or HAs at 4 to 5 years of age. Children with HAs had, however, somewhat better articulation skills. In their study, 25 children used HAs (M age at diagnosis 13.1 months, M age at HA fitting 14.4 months) and 26 children used CIs (M age at HL diagnosis 20.1 months, M age at CI activation 26.4 months). It is worth noting that the aforementioned studies have included children with mild-to-profound HL in the group of children fitted with HAs. This may be a confounding factor, since degree of HL and aided speech audibility contribute to spoken language outcomes (Wake et al. 2005; Stiles et al. 2012; Koehlinger et al. 2013; Tomblin et al. 2015). Children with profound HL may have gained little benefit from HAs at the time of the study. In addition, the ages at HL diagnosis were relatively high by today’s standards and children had mainly received unilateral CIs (Sininger et al. 2010; Fitzpatrick et al. 2011). During recent years, early bilateral cochlear implantation has become increasingly common in many developed countries for children with severe to profound HL (National Institute on Deafness and other Communication Disorders 2011). Bilateral CI use is shown to be associated with better sound localization acuity and speech perception ability in background noise (see Johnston et al. 2009 for review). Some reports already show that children with bilateral CIs have better spoken language abilities than children with unilateral CIs (Boons et al. 2012b, 2013; Sarant et al. 2014). Thus, we need more knowledge on spoken language skills of children with profound HL who have undergone early bilateral implantation and on children with mild-to-severe HL with early bilateral HA fitting.

In addition, we need continuous and systematic assessment on whether young children with mild-to-severe HL who use bilateral HAs develop age-appropriate spoken language and listening skills. For children with severe HL, such knowledge could even support implant candidacy criteria. For example, according to recent NICE-guidance (2019), severe to profound HL (i.e., qualification as poor enough hearing for CI candidacy) is defined as hearing only sounds louder than 80 dB HL at 2 or more frequencies (500, 1000, 2000, 3000, and 4000 Hz) bilaterally without acoustic HAs (in NICE-guidance 2009, a value of 90 dB HL was used). In the same guidance, an adequate benefit from acoustic HAs is defined as speech, language and listening skills appropriate to age, developmental stage and cognitive ability. This highlights the need for continuous evaluation of listening and spoken language skills of children with HAs in order to detect possible delays and support decision-making regarding implantation. Certainly, for such evaluation, norm-referenced and validated assessment methods are crucial. Namely, surveys on implant candidacy have shown differences in the interpretation of candidacy for children with significant residual hearing (Dettman et al. 2004; Fitzpatrick et al. 2009). Adults with severe HL in the 70- to 90-dB range commonly meet candidacy criteria for cochlear implantation, but in children these HL ranges can present difficult decisions for both parents (Hyde et al. 2010) and practitioners (Fitzpatrick et al. 2009). Recent studies also show that greater residual hearing before cochlear implantation is associated with greater rates of improvement in spoken language comprehension and expression (Niparko et al. 2010), and especially aided audibility with HAs, SII, is correlated with speech recognition performance in noise with CIs after implantation (Nickerson et al. 2019). Taken together, systematic evaluation of listening and spoken language skills is fundamental for development and implementation of auditory and language intervention strategies.

## THE PRESENT STUDY

The present study provides new knowledge on spoken language skills in children with mild-to-severe HL fitted with bilateral HAs and in children with profound HL who have received bilateral CIs. This study compares the language skills of children with bilateral HAs and bilateral CIs with normative values of age-peers with NH. In addition, the proportions of children with bilateral HAs or bilateral CIs below age-norms are compared with each other in order to investigate differences in the early language skills of these children. Herein, we focused on spoken language skills at the age of three years. It is recognized that 3-year-olds with NH can already master several language domains allowing for direct testing. Clinically, this age forms a valid point to assess any possible delays in spoken language development to prevent their long-term effects for later learning difficulties with the help of timely and targeted language intervention, if needed. We anticipate a positive effect on children’s spoken language skills based on early detection of HL through universal newborn hearing screening and fitting of bilateral HAs or bilateral CIs.

The present study also investigates auditory, child-related, and environmental factors associated with spoken language skills providing clinicians and parents with information to be utilized in implementing the best possible support for children with HL. Specifically, the aims of the present study were to examine spoken language comprehension, receptive and expressive vocabulary and phonological skills in Finnish children with bilateral HAs or with bilateral CIs at the age of 3 years. This study sought to compare the performance of the children with HL with the normative values of age-peers with NH, to analyze differences between the children with HAs and CIs, and to investigate factors associated with the outcomes. The present study gives a unique opportunity to investigate spoken language acquisition of children with HL in a language that differs greatly from the largely studied Germanic languages (i.e., a language with dominantly disyllabic or multisyllabic word forms and rich inflectional morphology; Karlsson 1999, Kunnari & Savinainen-Makkonen 2007; Kunnari et al. 2016). Only some reports exist on vocal development and early lexical development of Finnish children with bilateral CIs (e.g., Välimaa et al. 2018, 2019).

## MATERIALS AND METHODS

### Design

The current study analyzed spoken language skills of 3-year-old children with mild-to-severe HL who used bilateral HAs (BiHA group) and children with profound HL who used bilateral CIs (BiCI group). Children’s performance was compared to the age-norms of peers with NH and factors associated with spoken language skills were analyzed. The study was approved by The Ethical Committee of the Northern-Savonia Hospital District, Kuopio University Hospital, and it was in accordance with the tenets of the 1975 Declaration of Helsinki. Written consent was received from parents. The study is registered in the registry for Randomized Control Trials and Clinical Trials (ClinicalTrials.gov ID: NCT00960102).

### Background Information

Background information was collected with study-specific questionnaires directly from the parents/caregivers or with custom-made information forms from the patient files at university hospitals. Parents/caregivers reported information such as the birth weight of their child, any other disabilities experienced by their child, family history of HL and delayed language development/developmental language disorders, language(s) used at home, parental level of education, and active device use (devices used for more than 8 hours daily). The parental level of education was classified into seven levels according to the International Standard Classification of Education (1997). Level 1 refers to elementary school (grades 1–6). In Finland, compulsory education is nine years, which means that parents qualify at least as level 2 (comprehensive school grades 7–9). Upper secondary education qualifies as level 3 (senior high school/vocational training). Postsecondary/nontertiary education and the first stage of tertiary education qualify as level 4 (college level/community/junior college level). The second stage of tertiary education qualifies as level 5 (polytechnic degree or bachelor’s degree from a university), a higher academic degree qualifies as level 6 (master’s degree from a university), and researcher training as level 7 (advanced research qualification, i.e., licentiate or doctoral degree from a university). Data related to age of HL diagnosis, etiology, age at first HA fitting, age at active HA use, details on HA fitting procedures, age at CI activation, other health conditions and additional disabilities, and details regarding initiation of early family-centered speech and language therapy (early SLT) before the age of 2 years were collected from patient files.

### Participants

The participants comprised 56 children: 28 children in the BiHA group and 28 children in the BiCI group. The children came from a nationwide prospective multicenter follow-up study involving all five university hospitals in Finland. The national cohort of children with moderate-to-profound HL eligible for the study initially comprised 150 children (94 children eligible for the BiHA group and 56 children eligible for the BiCI group). Their HL was diagnosed through a combination of universal newborn hearing screening (applied since 2004) and Auditory Brainstem Responses or Auditory Steady state Responses at any of the university hospitals in Finland. The following exclusion criteria were applied to the study: Children fitted with HAs or implanted after the age of 2 years; children diagnosed with heart conditions, developmental brain disorders, chromosome anomalies, inner ear anomalies, impairments in both hearing and vision; conductive HL; and children whose parents both speak a language other than Finnish. Recruitment was successful for 82 children (43 children in the BiHA group and 39 children in the BiCI group). Because an accelerated prospective longitudinal design was implemented (i.e., children entered the study at various ages and were subsequently followed-up to the age of 6 years), all children assessed at the age of three years were included in the present study.

Overview of auditory, child-related and environmental factors and group-differences between the BiHA and the BiCI groups are presented in Table 1. The findings indicate no statistically significant differences between the groups in many of the auditory factors. The BiHA group was diagnosed at the mean age of 4.9 months and fitted with HAs at the mean age of 8.0 months. The BiCI group was diagnosed at the mean age of 4.3 months. All children in the BiCI group had at least a three-month HA trial before implantation (Nice 2009), after which they underwent cochlear implantation. Twenty-five children underwent simultaneous, and three children sequential, bilateral implantation. Mean age at CI activation was 13.1 months (range: 10–22). For the three children with sequential bilateral implantation, the second CI was activated at the mean age of 20.6 months (range: 18–23). Since age at CI activation affects the BiCI group’s hearing age, their hearing age at the chronological age of 3 years was 2.2 years, at most. Aided PTA_0.5–4 kHz_ measured at the age of three years indicated no statistically significant differences between the groups. Unaided PTA_0.5–4 kHz_ was not measured for the BiCI group. In addition, children’s speech perception was measured either as a word recognition score (i.e., percentage of words recognized correctly) or as a speech recognition threshold for words in dB HL (SRT_words_) (i.e., the dB HL level that yielded 50% correct responses). The mean aided word recognition score was 95% for the BiHA group (n = 8; SD = 7; range: 80–100) and 88% for the BiCI group (n = 15; SD = 12; range: 64–100). The mean unaided SRT_words_ was 49 dB HL for the children in the BiHA group (n = 14; SD = 18.6; range: 22–79).

**TABLE 1.** Overview of auditory, child-related, and environmental factors

Factors	BiHA (N = 28)	BiCI (N = 28)	Group-differences
Auditory factors			
Age, m: diagnosis, M (SD)	4.9 (3.2)	4.3 (2.5)	U* = 340, *p* = 0.38
Age, m: HA fitting, M (SD)	8.0 (4.5)	5.9 (2.2)	*U* = 281, *p* = 0.06
Age, m: active HA use†/CI activation, M (SD)	14.0 (9.6)	13.1 (2.5)	
Unaided PTA_0.5–4 kHz_, M (range: min–max), dB	53.8 (34–75)	nm	
Aided PTA_0.5–4 kHz_, M (SD), dB	38.7 (9.0)	27.5 (4.3)	*U* = 379, *p* = 0.82
Child-related factors			
Etiology			
Genetic (GJB2)	10 (7)	13 (13)	
Preterm	1	0	
Unknown	17	15	χ^2^‡ = 8.13, *p* = 0.23
Gender			
Male	18	14	
Female	10	14	χ^2^ = 1.12, *p* = 0.28
Chronological age, y;m, *M* (range: min–max),	3;1 (2;10–3;5)	3;1 (2;11– 3;11)	*U* = 365, *p* = 0.64
Additional disabilities			
Suspected delayed language	2	1	
Suspected attention deficit disorder/delayed language		1	Likelihood ratio = 0.23, *p* = 0.63
Environmental factors			
Parental hearing	Normal	Normal	
Native language	Finnish	Finnish	
Maternal level of education			
Low	9	5	
Medium	12	12	
High	7	11	χ^2^ = 2.03, *p* = 0.36
Parental involvement	Sufficient	Sufficient	
Early SLT	0	23	χ^2^ = 39.03, *p* < 0.001

* Independent samples Mann–Whitney U-test; *U* = test value; *p* = *p* value.

† At least 8 hours/day.

‡ Pearson Chi-Square test; χ^2^ = test value; nm = not measured; GJB2 = Mutation in the GJB2 (Connexin26) gene; low = levels 1–3, i.e., elementary and comprehensive school, and senior high school or vocational training; medium = levels 4 and 5, i.e., college, polytechnic or bachelor’s degree; high = levels 6 and 7, i.e., master’s, licentiate or doctoral degree from a university; Early SLT = initiation of family-centered speech and language therapy under the age of 2 years.

BiCI, children with bilateral cochlear implants; BiHA, children with bilateral hearing aids; *M*, mean.

published online ahead of print July 13, 2021.

Analysis of child-related factors showed no statistically significant differences in etiology, gender distribution, and chronological age at time of assessment, and in distribution of suspected additional disabilities (Table 1). A genetic deviance with the Connexin mutation (GJB2) caused HL in 25% of the children in the BiHA group and in 46% of the children in the BiCI group. The etiology remained unknown for approximately half of the children in the BiHA and the BiCI groups. In both groups, two children were suspected to have additional disabilities. Data regarding environmental factors showed that all children were living in a family with hearing parents and Finnish was the native language of at least one of the parents. Analysis of the distribution of maternal level of education classified as low (levels 1–3), medium (levels 4 and 5), or high (levels 6 and 7) indicated no group-differences. Parental involvement in the intervention process was rated as sufficient (i.e., according to expert ratings parents were motivated and/or able to fulfill their commitments, e.g., showing up for appointments, filling-in the questionnaires and participating in early SLT). However, there was a significant group-difference in the initiation of early SLT; none of the children in the BiHA group, but 23 children in the BiCI group, had received early SLT.

It is worth noting that all families had access to early hearing detection and intervention services at university hospitals (Joint Committee on Infant Hearing 2007; Joint Committee on Infant Hearing-Supplement 2013). They received access to service coordinators and support given by multiprofessional teams at least biannually/annually (e.g., medical doctors specialized in ear, nose, and throat diseases and voice, speech and language disorders; audiologists; speech and language therapists; psychologists; and social workers). However, the recommendation regarding appropriate audiologic and medical evaluations to confirm the presence of HL no later than at three months of age was met by only 20 children of the present study (36%), whereas 48 children (cumulative percentage 86%) were diagnosed by the age of six months and 53 children (cumulative percentage 95%) by the age of 7 months. The recommendation of HA fitting within one month of confirmed diagnosis was met by eight children in the BiHA group (29%) (range: <1–11 months) and by 16 children in the BiCI group (57%) (range: <1–4 months).

### Spoken Language Assessment

Children’s spoken language skills were evaluated with a comprehensive assessment battery focused on language comprehension, receptive and expressive vocabulary, and phonological skills. The tests were administered by the clinical or research speech and language therapists. During assessment sessions, children wore their bilateral HAs or bilateral CIs at their personal settings. All tests were standardized and norm-referenced on typically developing children. The assessment required approximately 1.5 to 2 hours, completed over one to two sessions. Play breaks were provided for the children as required to maintain attention and concentration. A semistructured test order was followed, but expressive vocabulary was assessed before receptive vocabulary and phonology to avoid learning effect. Sessions were video-recorded and scoring of tests was completed after test administration with the help of video recording.

#### Language Comprehension

The Reynell Developmental Language Scales, Third Edition (RDLS-III), Finnish version (Edwards et al. 1997) was used for assessment of spoken language comprehension. The RDLS-III comprises scales for receptive and expressive language. In the current study, the receptive Comprehension Scale was used. The Comprehension Scale is organized into nine sections: single words, relating two-named objects, agents and actions, attributes, noun phrases, locative relations, verbs, vocabulary and complex grammar, and inference skills. The child is asked to point to objects when the examiner names them, follow oral instructions of varying lengths or to point to pictures that match the given oral description. Raw scores were converted to standard scores. The scale provides norm-referenced scores, which are based on typical language levels for normally developing children with NH.

#### Receptive Vocabulary

The Receptive One-Word Picture-Vocabulary Test 4 (ROWPVT-4) Finnish version (Martin & Brownell 2010a) was used for assessment of receptive vocabulary. Test plates with four pictures are shown to the child. The child’s task is to point to or to identify the numbered picture that matches the word spoken by the examiner. Raw scores were converted to *z*-scores, which are based on typical receptive vocabulary scores for normally developing age-peers with NH. The *z*-score is measured in terms of SD from the mean; a *z*-score of 0 indicates that the score is identical to the mean score of the normative sample of age-peers, and a *z*-score of ±1.0 indicates a value that is 1 SD above or below the mean of the normative sample.

#### Expressive Vocabulary

The Expressive One-Word Picture-Vocabulary Test 4 (EOWPVT-4) Finnish version (Martin & Brownell 2010b) was used for assessment of expressive vocabulary. Expressive vocabulary is assessed by showing the child test plates with pictures. The child’s task is to verbally identify the picture. Raw scores were converted to *z*-scores based on typical expressive vocabulary scores for normally developing age-peers with NH.

#### Phonological Skills

The Test of Phonology: Assessment of children’s phonological skills (Kunnari et al. 2012) was used for evaluation of phonological skills. In this test, the child is shown pictures and asked to verbally identify them. The child’s responses are phonemically transcribed and scored after administering the test. Scoring is based on phonological skills. This means that phonetic errors in articulation are not taken into account in scoring, but are marked on the test form as additional information. Test scores can be given for total score in phonological skills, and in subtest scores in phonotactic skills (word, syllable and word length, and phoneme sequences) and paradigmatic skills (phoneme inventory). Raw scores can be converted into percentile ranks, which are based on normal phonological skills for normally developing age-peers with NH. Percentile ranks define the percentage of children with a corresponding or lower score compared to the normative sample (Maxwell & Satake 2006). Percentile rank 16 (raw score 46) corresponds to the value that is 1 SD below the mean of the normative sample, and percentile rank 84 (raw score 60) corresponds to the value that is 1 SD above the mean.

### Statistical Analysis

#### Language Skills and Group-Differences in Proportions of Children Below Age-Norms

Primary outcome measures were spoken language comprehension, receptive and expressive vocabulary, and phonological skills. Means and SDs were calculated for the outcome measures. The proportion of children within ± 1 SD of the mean of the normative sample of children with NH, and at least 1 SD above or below the mean was calculated. Odds ratio (OR) and 95% confidence interval (CI) were calculated to analyze group-differences (BiCI; BiHA) between the proportions of children at least 1 SD below the mean of the normative sample. The BiCI group was used as a reference.

#### Factors Associated With Language Skills

Means and SDs were reported for continuous variables (e.g., spoken language tests) and numbers and percentages for categorical variables (e.g., gender and maternal level of education). All data sets were normally distributed (Kolmogorov–Smirnov, *p* > 0.05). Variables associated with spoken language skills were modeled with the analysis of covariance (ANCOVA) models separately for the BiHA and the BiCI groups, because some of the factors that might predict children’s performance could be different between the groups (e.g., unaided PTA_0.5–4kHz_, age at CI activation). The variables included in the study were grouped into auditory, child-related, and environmental factors. Multiple ANCOVA models were constructed separately for each language measure. The same group-specific factors were used in the analyses across all language domains. For the BiHA group, auditory factors were age at HL diagnosis, age at first HA fitting, age at active HA use (≥8 hours daily) and unaided PTA_0.5–4 kHz_. For the BiCI group, auditory factors were age at HL diagnosis and age at activation of CIs. For both groups, gender (boy and girl) served as a child-related factor and maternal level of education (low; medium; and high) as an environmental factor. Bonferroni adjustment for multiple comparisons was used in post hoc analysis of the effect of maternal level of education. Estimated marginal means (EMM) and 95% CI were modeled for the categorical variables. Parameter estimates with 95% CI were modeled for the continuous variables (covariates) to analyze the relationship between covariates and dependent variables (i.e., spoken language tests). Effect sizes were determined by computing the partial eta squared (*η*_*p*_^*2*^, Olejnik & Algina 2003), which indicates the variance accounted for by the factor plus the residual error of the model. Throughout the Results section, effect sizes are reported using the set of conventions defined by Bakeman (2005): *small* equals 0.02, *medium* equals 0.13, and *large* equals 0.26.

## RESULTS

### Spoken Language Comprehension

**Fig. 1.**
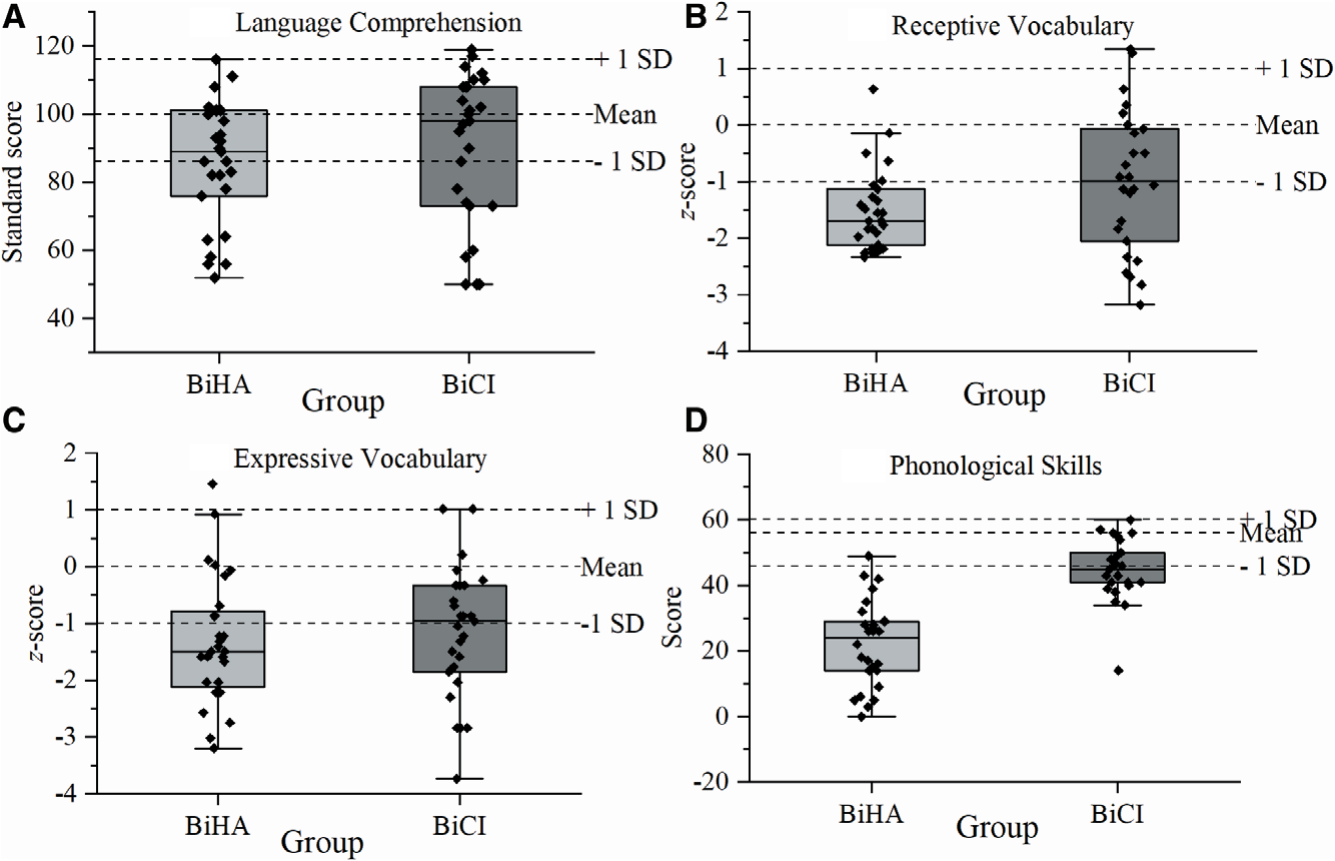
Spoken language skills in children with bilateral hearing aids (BiHA group) and bilateral cochlear implants (BiCI group): spoken language comprehension (A), receptive vocabulary (B), expressive vocabulary (C), and phonological skills (D). The box plots represent the smallest observation, lower quartile, median (bold line), upper quartile, largest observation. Individual data are plotted on the box plots. The dashed lines represent test-specific mean and ±1 SD from the mean of the normative sample.

Means and SDs in children’s language skills, and proportions of children within ±1 SD and at least 1 SD above or below the mean of the normative sample of 3-year-old age-peers with NH are presented in Table 2. Figure 1 shows the group-data in all language tests and the individual data is plotted to visualize the distribution. In spoken language comprehension (Fig. 1A; Table 2), one half of the children in both groups scored within ±1 SD of the mean of the normative sample of 3-year-old children with NH. Approximately one half of the children in the BiHA group and one-third of the children in the BiCI group scored 1 SD or more below the mean of the normative sample.

**TABLE 2.** Means (M) and SD in children’s spoken language skills, and proportions of children at least 1 SD above the mean of the normative sample, within ± 1 SD, and at least 1 SD below the mean

Spoken Language Test	Group	*M* (*SD*)	Proportion (%) > +1 *SD*	Proportion (%) Within ± 1 *SD*	Proportion (%) < −1 *SD*	OR	95% CI [Low, High]
Language comprehension	BiHA	86 (18.0)	3	56	41	1.4	[0.454, 4.17]
	BiCI	92 (21.7)	11	56	33		
Receptive vocabulary	BiHA	−1.5 (0.7)	—	18	82	**4.4**	**[1.28, 15.18]**
	BiCI	−1.0 (1.3)	8	42	50		
Expressive vocabulary	BiHA	−1.4 (1.1)	4	28	68	2.3	[0.76, 6.80]
	BiCI	−1.2 (1.2)	7	45	48		
Phonological skills	BiHA	22 (13.0)	—	4	96	**18.0**	**[2.10, 154.58]**
	BiCI	45 (9.7)	4	36	60		

OR and 95% CI indicate group-differences (OR indicates the odds [likelihood] for poorer spoken language skills in the BiHA group).

BiCI group used as reference in the analyses of the group-differences;

Bold font indicates significant differences between the groups.

BiCI, children with bilateral cochlear implants; BiHA, children with bilateral hearing aids; CI, confidence interval; OR, odds ratio.

### Receptive and Expressive Vocabulary

In receptive vocabulary, approximately one-fifth of the children in the BiHA group and one half of the children in the BiCI group scored within ± 1 SD of the mean of the normative sample (Fig. 1B; Table 2). In the BiHA group, a majority of the children scored 1 SD or more below the normative mean compared to one half of the children in the BiCI group. In expressive vocabulary, one-third of the children in the BiHA group and almost one half of the children in the BiCI group scored within ± 1 SD of the mean of the normative sample (Fig. 1C; Table 2). In the BiHA group, two-thirds of the children scored 1 SD or more below the normative mean compared to one half of the children in the BiCI group.

### Phonological Skills

The present study showed that especially phonological skills seemed to induce difficulties for the children in the BiHA group; only a fraction of the children in the BiHA group scored within ± 1 SD of the mean of age-peers with NH compared to over one-third of the children in the BiCI group (Fig. 1D; Table 2). Taken together, the present study showed that especially receptive vocabulary and phonological skills induced difficulties for the children in the BiHA and the BiCI groups at the age of 3 years.

The difference in the proportions of children in the BiHA and the BiCI groups at least 1 SD below the mean of the normative sample are demonstrated by OR, which indicates the odds (likelihood) for poorer spoken language skills in the BiHA group (Table 2). The BiCI group was used as a reference. The results showed that the children in the BiHA group were more likely to have poorer receptive vocabulary and phonological skills than the children in the BiCI group. For language comprehension and expressive vocabulary, the ORs were around 1 indicating no significant differences between the groups.

### Factors Associated With Spoken Language Skills

#### The BiHA Group

The ANCOVA model revealed that there was a significant effect of unaided PTA_0.5–4 kHz_ on spoken language comprehension of children in the BiHA group with a large effect size (*η*_*p*_^*2*^ = 0.32) (Table 3). Parameter estimates indicated that a 1 dB decrease in unaided PTA_0.5–4 kHz_ (i.e., better hearing level) improved spoken language comprehension by 1.7 points in standard scores (95% CI [0.39, 3.00]). However, none of the other factors (age at HL diagnosis, age at HA fitting, age at active HA use, gender, maternal level of education) modeled in the present study predicted the spoken language skills of the children in the BiHA group. Taken together, these results suggest that better unaided hearing has a positive effect on spoken language comprehension. The results also suggest that other variables than the ones measured here may be more sensitive and may better predict spoken language skills for children with relatively early diagnosed mild-to-severe HL, who have been fitted with bilateral HAs early, and who do not have clear additional disabilities.

**TABLE 3.** Analysis of covariance models (ANCOVA) for children with BiHA group

Between-Subjects Factors	Language Comprehension				Receptive Vocabulary				Expressive Vocabulary				Phonological Skills			
	*F*	*df*	*p*	*η*_*p*_^*2*^	*F*	*df*	*p*	*η*_*p*_^*2*^	*F*	*df*	*p*	*η*_*p*_^*2*^	*F*	*df*	*p*	*η*_*p*_^*2*^
Age, m: diagnosis	1.52	1	0.24	0.09	0.18	1	0.68	0.02	0.19	1	0.67	0.01	0.35	1	0.56	0.02
Age, m: HA fitting	0.58	1	0.46	0.04	0.01	1	0.92	0.00	0.05	1	0.83	0.01	1.79	1	0.20	0.10
Age, m: active HA use	0.05	1	0.83	0.00	0.05	1	0.82	0.00	0.63	1	0.44	0.04	0.04	1	0.84	0.00
Unaided PTA_0.5–4 kHz_	**7.61**	**1**	**0.01**	**0.32**	2.27	1	0.15	0.12	1.97	1	0.18	0.11	0.15	1	0.71	0.01
Gender	0.09	1	0.76	0.01	0.20	1	0.66	0.01	0.77	1	0.39	0.04	0.20	1	0.83	0.02
Maternal education	1.07	2	0.37	0.12	0.28	2	0.76	0.03	1.14	2	0.34	0.12	0.13	2	0.88	0.02

Bold font indicates the significant effect.

*η*_*p*_^2^, partial eta-squared effect size (*small* equals 0.02, *medium* equals 0.13, and *large* equals 0.26); BiHA, children with bilateral hearing aids; *df*, degrees of freedom; *F*, test value; Unaided PTA_0.5–4 kHz_ dB HL, unaided pure-tone average over the frequencies of 0.5–4 kHz.

#### The BiCI Group

For the BiCI group, there was a significant effect of maternal level of education on spoken language comprehension with a large effect size (*η*_*p*_^*2*^ = 0.30) (Table 4). Post hoc analysis with Bonferroni adjustment for multiple comparisons showed that the children whose mothers had a high level of education (i.e., 6 and 7; n = 11) performed significantly better on language comprehension than the children whose mothers had a low level of education (i.e., 2 and 3; n = 5) (EMM difference 29 points in standard scores, *p* = 0.03). The maternal level of education also had a significant effect on expressive vocabulary with a large effect size (*η*_*p*_^*2*^ = 0.53). Post hoc analysis with Bonferroni adjustment for multiple comparisons showed a similar trend as in language comprehension; the children whose mothers had a high level of education performed significantly better on expressive vocabulary than the children whose mothers had a low level of education (EMM difference 1.85 points in *z*-sores, *p* = 0.001). In phonological skills, there was a statistically significant effect of gender with a medium effect size (*η*_*p*_^*2*^ = 0.20) showing that girls performed better than boys (EMM difference 8 points, *p* = 0.04).

**TABLE 4.** Analysis of covariance models (ANCOVA) for children with BiCI group

Between-Subjects Factors	Language Comprehension				Receptive Vocabulary				Expressive Vocabulary				Phonological Skills			
	*F*	*df*	*p*	*η*_*p*_^*2*^	*F*	*df*	*p*	*η*_*p*_^*2*^	*F*	*df*	*p*	*η*_*p*_^*22*^	*F*	*df*	*p*	*η*_*p*_^*2*^
Age, m: diagnosis	0.00	1	.099	0.00	3.02	1	0.10	0.13	**7.82**	**1**	**0.01**	**0.27**	1.55	1	0.23	0.08
Age, m: CI activation	3.37	1	0.08	0.14	2.78	1	0.11	0.12	**5.05**	**1**	**0.04**	**0.19**	0.04	1	0.84	0.00
Gender	0.41	1	0.53	0.02	2.63	1	0.12	0.12	0.29	1	0.60	0.01	**4.73**	**1**	**0.04**	**0.20**
Maternal education	**4.40**	**2**	**0.03**	**0.30**	3.53	2	0.05	0.26	**11.82**	**2**	**<0.001**	**0.53**	2.55	2	0.10	0.21

Bold font indicates the significant effect.

*η*_*p*_^2^, partial eta-squared effect size (*small* equals 0.02, *medium* equals 0.13, and *large* equals 0.26); BiCI, children with bilateral cochlear implants; *df*, degrees of freedom; *F*, test value.

When looking at the effect of the covariates, the data showed that age at HL diagnosis and age at CI activation had a significant effect on expressive vocabulary with a large or medium effect size (*η*_*p*_^*2*^ = 0.22 and *η*_*p*_^*2*^ = 0.19, respectively) (there was a positive relationship between the two covariates and expressive vocabulary). Taken together, the results suggest that a high maternal level of education predicts spoken language comprehension and expressive vocabulary for children with bilateral CIs. In addition, age at HL diagnosis and age at CI activation seemed to predict expressive vocabulary skills and gender predicted phonological skills.

## DISCUSSION

This study examined spoken language skills in 3-year-old early diagnosed children with mild-to-severe HL who used bilateral HAs (BiHA group) and children with profound HL who used bilateral CIs (BiCI group). Proportions of children at least 1 SD below the mean of the normative sample of age-peers with NH were analyzed and possible group-differences were explored. In addition, auditory, child-related, and environmental factors associated with outcomes were analyzed.

### Spoken Language Skills

The present study showed that especially phonological skills and receptive and expressive vocabulary skills induced difficulties for the children in the BiHA group at the age of three years. For over 90% of children in the BiHA group, phonological skills were 1 SD or more below the mean of the normative sample of age-peers with NH. In receptive vocabulary, the proportion was somewhat lower, but still high (80%). Spoken language comprehension seemed to be the least vulnerable, but still almost one half of the children in the BiHA group were 1 SD or more below the normative mean. For the children in the BiCI group, phonological skills and receptive vocabulary were the most difficult followed by expressive vocabulary and spoken language comprehension. Approximately one half of the children in the BiCI group performed 1 SD or more below the mean of the normative sample. Our findings on the high proportion of children with HL being below age-norms confirm earlier studies showing that children with HL seem to lag behind their peers with NH at the age of three years in several language domains (e.g., Duchesne et al. 2009; Fitzpatrick et al. 2011; Boons et al. 2012a; Ching et al. 2013; Tomblin et al. 2015).

The present study further showed that especially acquisition of phonological skills and receptive and expressive vocabulary seem to be language domains that are highly vulnerable for children with HL. This finding is in keeping with existing literature showing that children with HL, both HA and CI users, often have lower phonological skills (Baudonck et al. 2010; Baudonck et al. 2011) and lexical skills compared to children with NH in childhood (e.g., Duchesne et al. 2009; Fitzpatrick et al. 2011; Lund 2016; Välimaa et al. 2018; Wie et al. 2020). This has been verified in many languages. We were, however, concerned that approximately one-third of the children in both groups were 1 SD or more below age-norms in spoken language comprehension at the age of three years, and that the percentage was even higher in lexical and phonological skills. Based on earlier research, we would have expected this proportion to be lower, because the children were fitted with bilateral HAs relatively early (M = 8 months, SD = 4.5) or had received bilateral CIs early (M = 13.1 months, SD = 2.5) and the majority of the children did not have any diagnosed additional disabilities. The value 1 SD or more below the mean of the normative sample of age-peers with NH forms a risk criterion for less favorable spoken language development at each language domain (i.e., language comprehension, lexical, and phonological skills). The criterion clearly shows the proportion of children with poorer language skills for whom close monitoring of the adequate benefit from HAs (i.e., aided audibility, listening, speech perception, and spoken language skills) and CIs (i.e., listening and speech perception and spoken language skills) and the need for modification of intervention implementation has to be considered. The fact that many children with HL lag behind their age-peers with NH in spoken language skills highlights that, despite newborn hearing screening, early bilateral HA fitting, or bilateral implantation, HL is still a risk factor for age-appropriate development of spoken language skills in early childhood (e.g., Wake et al. 2005; Boons et al. 2012b; Ching et al. 2013; Tomblin et al. 2015).

The present study also showed that some children in both groups performed within ± 1 SD of the mean and some even 1 SD or more above the mean of the normative sample of age-peers with NH. Thus, our findings are in concordance with the many previous studies having shown the great individual variability in the spoken language skills of children with HL (Boons et al. 2012a; Tomblin et al. 2015; van Wieringen & Wouters 2015). Our results also confirm earlier findings showing that children who are diagnosed and fitted with HAs, or undergo implantation early and have no additional disabilities, have a better opportunity to achieve age-appropriate spoken language development compared to later identified children with HL who have received HAs or CIs later (Yoshinaga-Itano et al. 2010; Boons et al. 2012a; Fulcher et al. 2012; Dettman et al. 2016; Yoshinaga-Itano et al. 2018). Taken together, the present results give us a first impression of difficulties in lexical and phonological skills at the age of three years and shows that we cannot anticipate optimal language development in children with early diagnosis and early HA fitting or bilateral implantation. In every healthcare system, children with HL need to be systematically assessed because only in this way can family support and speech and language intervention be offered. Spoken language delay at the age of 3 years may bear long-term consequences for literacy and learning (Geers et al. 2008; Johnson & Goswami 2010). It can also be an indicative of additional disabilities such as autism spectrum disorder, neurological and developmental disorders, or developmental language disorder (Hawker et al. 2008; Boons et al. 2013; Ching et al. 2013; de Hoog et al. 2015).

Furthermore, the present study brings highly needed information about children with HL who are acquiring Finnish, a language with mainly disyllabic and multisyllabic word structure and very rich inflectional morphology (i.e., 15 cases for nouns and person, number, tense, mood, and voice inflection for verbs) (Karlsson 1999; Kunnari & Savinainen-Makkonen 2007; Kunnari et al. 2016). Only some reports exist on vocal development and early lexical development of Finnish children with bilateral CIs (e.g., Välimaa et al. 2018, 2019). The fact that nearly one half of the children with HL had spoken language comprehension skills 1 SD or more below the mean of the normative data indicates that the rich inflectional system in Finnish, that is, suffixes, may be difficult for a child with HL to perceive and this might affect spoken language comprehension. The suffixes are located at the end of words, where the sound pressure level is often lower, due to the falling intonation typical of Finnish. Yet, these suffixes are critical to the meaning of sentences. On the word level, variability in the syllable structures and multisyllabic words (i.e., 3- to 4-syllable words) may affect acquisition of phonological and lexical skills. These language-specific features warrant more detailed future analyses of the acquisition of spoken language in children with HL, for example, analysis of the association between spoken language comprehension and production of phonetic and morphosyntactic structures.

### Group-Differences

This study showed clear group-differences in spoken language skills of children in the BiHA and the BiCI groups. The children in the BiHA group were more likely to have poorer receptive vocabulary and phonological skills than the children in the BiCI group. Spoken language comprehension and expressive vocabulary showed a similar tendency. The finding that having CIs had a positive effect on receptive vocabulary and phonological skills is consistent with the study by Sininger et al. (2010). In their study, CI use was associated with better receptive and expressive language outcomes in preschool as measured with RDLS compared to children with mild-to-profound HL who use HAs. In the present study, the BiCI group was more likely to perform better, even though the BiHA group did not include children with profound HL.

In addition to CI use, Sininger et al. (2010) pointed out the large variation in the amount and type of auditory intervention children received, which may also have contributed to their results. Our finding on the better performance of the BiCI group compared to the BiHA group on spoken language skills at the age of 3 years is in contrast to a study by Fitzpatrick et al. (2011). They found no significant differences between children with severe to profound HL who used CIs or HAs at four to five years of age in receptive and expressive language skills. In their study, all children received a similar type and quality of auditory-focused intervention, independent of whether they had less severe HL and received HAs or had more severe HL and received unilateral CIs. It is worth noticing that at the chronological age of three years, the hearing age of the BiCI group was approximately 2 years (M age at CI activation 13.1 months), yet they outperformed the children in the BiHA group in receptive vocabulary and phonological skills. The findings of our study highlight the impact of access to auditory information early in life. There is vast development in early speech perception skills, language-specific phonological representations and segmentation skills during infancy in children with NH (Kuhl 2004). Taken together, it is important to continue assessing children’s spoken language skills in longitudinal prospective design in order to explore whether the children with HL catch-up to their peers with NH before they begin school or whether they continue to lag behind or even fall further behind the peers with NH in some language domains (see e.g., Boons et al. 2012a; Tomblin et al. 2015; Wie et al. 2020).

When looking at the differences between the BiHA and the BiCI groups in spoken language skills in our study, some important aspects related to the BiHA group need to be addressed. First, for the BiHA group, there was a clear delay between the first HA fitting and patient file records of systematic HA use. Obviously, this factor may contribute to the lower performance of the BiHA group. Recently, Walker et al. (2013, 2015) showed that families may be dealing with HA compliance issues and that child-related challenges may cause less-than-optimal HA use consistency. Fortunately, these challenges may decrease by the age of 2 years (Walker et al. 2013). Parents may also be aware that individual differences in language acquisition are greater for younger children than for older children (Lyytinen 1999; Fenson et al. 2007), and therefore, they are not concerned about their child’s language acquisition. Thus, they may not recognize the beneficial effect of daily systematic HA use for language development of younger children. In addition, children with mild-to-moderate HL may still have some useful level of hearing without their HAs. They may function actively in daily living, and poor speech perception or spoken language skills may be hidden by a child’s active participation and functioning. Good auditory speech perception is widely recognized as fundamental for spoken language development and phonological and lexical processing, and inconsistent HA use has been shown to negatively affect spoken language development of children with HL (Walker et al. 2015a). In addition, recent research has shown that full-time CI use (i.e., > 8 or 9 hours daily) is not necessarily established for all children with CIs by the age of three years (Easwar et al. 2016; Bush et al. 2017; Park et al. 2019). Yet, full-time CI use is associated with better spoken language skills at the age of three years (Park et al. 2019). Therefore, we recommend that parents are continuously reminded of the benefits of systematic daily HA and CI use, regardless of the degree of HL.

Second, it is important to consider the clear difference between the BiHA and the BiCI groups in implementation of early SLT (i.e., individual SLT starting below the age of 2 years). Only three children in the BiHA group received early SLT, compared to 23 out of 28 children in the BiCI group. The aforementioned finding needs to be considered in detail in relation to our national healthcare system. In Finland, children with severe to profound HL who receive CIs more often also receive early SLT due to the obvious risk factor of severe to profound HL for spoken language development. Bearing in mind that over 95% of children with HL are born to families with NH (Mitchell & Karchmer 2004) and no sign language skills, spoken language development is one of the main expectations of the parents after CI activation. Thus, early SLT is often implemented before or directly after CI activation. For children with mild-to-severe HL who receive HAs, individualized SLT may be implemented later, if needed, but the decision is often based on suspicion or clear signs of spoken language delay. SLT has been found to be highly cost-effective (Law et al. 2004), and early language support and intervention may decrease the long-term effects of HL for spoken language development, literacy, and academic skills. The findings of this study on the likelihood of poorer spoken language skills in the BiHA group compared to the BiCI group, clearly highlight the need for healthcare providers to consider early SLT for all families with children with HL, especially if any doubts arise on systematic HA use, the quantity, and quality of parental language use in daily communication. The findings inevitably warrant questioning of whether group-differences would have been less distinct if similar opportunities for early SLT had been provided for the groups. Without doubt, this has to be considered in relation to national resources in hearing care and multiprofessional teams.

### Factors Associated With Spoken Language Skills

The present study showed that for the BiHA group, only unaided PTA_0.5–4 kHz_ was associated with spoken language comprehension. As such, this finding is in keeping with previous studies showing that the more severe the HL, the poorer the spoken language outcomes (e.g., Tomblin et al. 2015). In the present study, none of the other variables (age at diagnosis, age at HA fitting, age at active HA use, gender, and maternal level of education) was shown to significantly affect spoken language skills. The fact that age at HL diagnosis and age at HA fitting did not predict children’s spoken language skills in the present study is consistent with some of the recent studies showing either a weak or no association between these variables and spoken language skills (e.g., Ching et al. 2013; Tomblin et al. 2015). In our study, all the children in the BiHA group were diagnosed with HL and fitted with HAs relatively early (M = 4.9 months and M = 8.0 months, respectively), which may diminish the effect of early diagnosis and HA fitting compared to the era before newborn hearing screening, when children were diagnosed later in toddlerhood.

In the BiCI group, age at HL diagnosis and age at CI activation had a significant effect on expressive vocabulary. This is in keeping with numerous studies showing the beneficial effect of early HL diagnosis and implantation for spoken language skills of children with CIs (Boons et al. 2012a; Geers et al. 2016; Dettman et al. 2016; Yoshinaga-Itano et al. 2018; Wie et al. 2020). For all children in this study, CIs were activated before the age of 2 years (M = 13.1 months), which can be considered as a relatively young age at activation. The fact that it still showed a clear effect on spoken language skills highlights the need for early CI activation. It seems reasonable to understand the beneficial effects of early access to auditory information in light of fundamental development in early speech perception skills, language-specific phonological and lexical representations and segmentation skills during infancy (Kuhl 2004).

In the present study, we found an effect of gender in the BiCI group, with girls having better phonological skills. This is in line with a previous result by Ching et al. (2013), who showed that girls had better global language outcomes. In their study, global language outcome included several receptive and expressive language tests. There are studies, however, showing no gender differences in early spoken language development of children with CIs (Boons et al. 2012a; Yoshinaga-Itano et al. 2018). The present study also showed a significant effect of maternal level of education. The children in the BiCI group whose mothers had a high level of education had better spoken language comprehension and expressive vocabulary skills. This is consistent with a number of previous studies that have shown the advantageous effect of high maternal level of education on spoken language both in children with NH and in children with HL (Niparko et al. 2010; Reilly et al. 2010; Ching et al. 2013; Yoshinaga-Itano et al. 2018; Wie et al. 2020). This advantageous effect is possibly related to the associations between high maternal level of education and quantity and quality of parental linguistic input (Vohr et al. 2013; Ambrose et al. 2015). Thus, healthcare providers should be aware of the family’s level of education and offer systematic and continuous counseling and coaching related to good-quality parental language use to all parents of children with HL. This can be provided, for example, by qualified SLTs. The effect of poor-quality and sparse spoken language input may be exacerbated by poor speech perception skills as a consequence of HL.

### Limitations and Future Directions

There are several limitations in this study that need to be addressed. The present findings are applicable only to children with HL at three years of age and should not be generalized to other age groups. There is evidence that children with HL may narrow or close the gap with NH peers as they grow (Connor et al. 2006; Duchesne et al. 2009; Tomblin et al. 2015; Wie et al. 2020). On the other hand, there is also evidence that after initial closing, the gap could later reappear for some language domains (e.g., receptive vocabulary; Wie et al. 2020). As a future direction, the children will be assessed at the ages of 4, 5, 6, and 10 years in a prospective longitudinal design. However, the findings of this study point out the need for early assessment and considerations on changes in the implementation of early intervention (e.g., early SLT) to avoid long-term effects of HL and spoken language delay of children’s spoken language development.

The fact that the present study used test-specific normative data of age-peers with NH may also be considered a limitation. Using a control group instead of normative data could have made it possible to match the groups of children with HL and with NH in order to ensure exactly the same SES level in both groups. There is evidence that more thorough matching of the children with HL and NH could reveal even greater differences between these children (Tomblin et al. 2015). In the present study, the maternal level of education was used as a proxy for SES. The cumulative percentage of mothers with medium or high level of education was higher than in Finnish women aged 25–44 years, on average (Official Statistics of Finland 2021). Since study participation is always based on voluntary consent, the families with higher level of education or SES may also be more active in test standardization and norm-referencing inducing a threat for sample diversity (see e.g., Edwards et al. 1997; Kunnari et al. 2012; Edwards et al. 2011). Thus, a sample of children with HL with greater diversity could perform poorer than the children in the present study (see e.g., Moeller et al. 2015). In the future, detailed studies of children with greater diversity are needed. However, when children with HL are compared to their NH age-peers in terms of normative values, it is possible to utilize the normative mean, and the values ± 1 SD of the mean of the normative data, to indicate a risk criterion for less favorable spoken language development which can be utilized in (re)consideration of intervention needs.

Another limitation lies in the fact that we did not collect data on the exact hours of daily HA use and aided audibility with HAs. According to parental reports, all children with HAs used their devices for at least eight hours daily. In the future, it would be important to collect exact data on daily HA use, as well as on daily CI use, for example, with the help of data logging systems, since systematic device use has been reported to promote spoken language development (Walker et al. 2015a; Easwar et al. 2016; Busch et al. 2017; Park et al. 2019). In addition, aided speech audibility, SII, could have provided insights into its potential effect on spoken language outcomes. Previous studies have shown that aided speech audibility may be below the average of the normative range for the SII based on the degree of HL for children with HAs (e.g., McCreery et al. 2015). This has also been shown to affect spoken language outcomes (Stiles et al. 2012; Tomblin et al. 2015). In Finland, aided speech audibility is not frequently assessed in clinical practice, thus no systematic information is gained about the actual HA fitting quality. In the future, it will be essential to conduct real ear measurements or real ear-to-coupler-difference measurements to investigate the quality of HA fitting, and to calculate aided speech audibility of children with HAs already during preschool years to ensure good-quality HA fitting. Such information is important also for implant candidacy criteria (see e.g., NICE 2019).

Our study also revealed that speech perception skills of children with HL were not systematically assessed below and at the age of 3 years. Speech perception is closely related to spoken language development (Wie et al. 2020) and therefore speech perception needs to be systematically assessed. At the moment, there are no carefully validated and norm-referenced speech perception tests in silence and in noise for children in Finland, although these are under development (Välimaa et al. 2017; Willberg et al. 2020). This certainly contributes to of the infrequency of speech perception assessment. In the future, it will be important to move away from assessing unaided and aided audiometric thresholds only, since children may have differing speech perception skills with the same PTA.

Although the effect of seven auditory, child-related, and environmental factors on spoken language skills were explored in this study, evidently some factors remain unknown. For example, it is important to analyze quality and quantity of parental spoken language input to children with HL and parent-child interaction, because these have been found to play a crucial role in spoken language development of children with HL (e.g., Szagun & Stumper 2012; Nittrouer et al. 2020). In this study, all children with HL had access to early hearing detection and intervention services and data was collected of the age at implementation of early SLT. However, we were not able to count exact dosage or contents of SLT and utilize it as an independent variable in the analyses. Such factors need to be addressed in the future.

## CONCLUSIONS

The present study joins others in showing that a substantial number of young children with HL still lag behind their peers with NH, especially in receptive and expressive vocabulary and phonological skills, despite implementation of universal newborn hearing screening and bilateral HA fitting or bilateral implantation at an earlier age. Thus, our study provides evidence that assessment of spoken language in all levels of language (i.e., phonology, vocabulary, morphology, and syntax) is important. Our study also provides evidence that children with mild-to-severe HL using bilateral HAs may be more likely to have poorer spoken language skills than children with profound HL using bilateral CIs, especially if children with HAs do not receive similar opportunities for early intervention as children with bilateral CIs. Close exploration of spoken language skills of all children with HL is important. Only this way can intervention be allocated correctly.

Our findings corroborate those of several studies indicating that high maternal level of education is associated with better spoken language development. Higher levels of parent education may result in better spoken language skills, possibly because higher education is associated with good-quality of caregivers’ linguistic input (Vohr et al. 2013; Ambrose et al. 2015). Thus, healthcare providers should be aware of the family’s level of education and offer counseling and coaching related to good-quality parental language use to all parents of children with HL. To conclude, these findings clearly point out the need for healthcare providers to offer all parents counseling and support regarding good-quality parental language input. Furthermore, it is important to consider each child with severe to profound HL individually with regard to HA benefit, possibility of (bilateral) implantation and early SLT, especially if any doubts arise related to HA benefit, quantity, and quality of parental language use or delay in spoken language development.

## ACKNOWLEDGMENTS

The authors thank Dr. Eila Lonka for collaboration and for her constructive comments on the earlier stages of the current study. The authors are deeply grieved that she was not able to participate as a coauthor.
